# American System of Medical Education

**Published:** 1857-08

**Authors:** 


### American System of Medical Education.

[Communicated for Iowa Medical Journal.]

*Messrs. Editors:*—The recent action of the American Medical Association, regarding the prevailing system of Medical Education, has induced renewed activity on the part of those who have a particular mania for working great reforms and being looked upon as great reformers. In the west, where it is of course expected there can be no GREAT MEN, but only monkeyish imitators of those above us, it is perhaps hardly proper that we should have any aspirations towards the fame of aiding to bring about the medical millennium soon to be ushered in, but it may be permitted us to talk of what our betters are doing, and (among ourselves of course,) find a little fault with them when we are displeased with what they say and do.

In the July number of that most excellent journal, the North American Medico-Chirurgical Review, I notice an article, by a contributor, in which the "American System of Medical Education" is considered at some little length—its faults fully shown up and its virtues, (the writer found none after the first paragraph,) freely eulogized.

This writer condemns the custom, prevalent in a few schools, of giving two courses of lectures per annum—the lecturing in two or more different schools by one professor—too scanty corps of professors, and the want of sufficient clinical instruction.

The first he denounces as a cunning device of the adversary, whereby colleges are able to cheat, as to time. The second he considers the fruit of self conceit of the "revolving professor," and directly injurious to the schools in which he officiates, by dividing and neutralizing the teachers' influence.

The third he esteems a sin against the general principle, that the greatest productiveness is secured by a division of labor.

The fourth he considers decidedly the most serious fault of the whole system, and here he *asserts* so dogmatically, I am forced to believe he is a lecturer upon clinical medicine in some of the schools. He pronounces it "absurd to argue this question," and I agree with him; but we might perhaps be found to differ widely as to what constitutes clinical instruction. He would tell you that clinical instruction consists in walking through the wards of a hospital, examining and prescribing for patient after patient, with a few remarks upon the condition &c., of each, and this with a large number of students at the professor's back, of whom, perhaps, one in ten can, by the hardest, catch a glimpse of a patient's face. He will tell you this, not in words as I now do, but by referring you for his definition of the term, to the course pursued in some of the schools.

But I go further than he and claim that students must have instruction at the bed-side and in such a way that they may themselves examine (under instruction,) patients, and be taught to detect the symptoms of disease, the signs of pathological conditions, and the indications to be fulfilled in efforts to cure. I would repeat, in this connection, what the writer says at the commencement of his article: "Excellence of plan and faultiness of detail are by no means incompatible." Indeed, as to the plan and detail of clinical instruction, in any true and useful sense, I think the compatibility of the two is most fully proven by the experience of a large majority of the students of our *larger*, and (per consequence,) *better* schools. There has been, and is, a great noise, and but little *substance* in the matter as now managed outside of the opportunity it gives the schools, having "larger hospital advantages," to cheat, not "in time," but in offering hypothetical inducements to students to attend their own, rather than other schools. I do not know if some plan may be fixed upon to overcome the difficulties in the way of making clinical instruction in the schools what it ought to be. I hope so, most sincerely, for now I do know, that to four out of five of students, it is (outside of the surgical operative clinique,) one of the most arrant humbugs of a humbuging age. I wish to be understood here, Messrs. editors, as referring to our most popular schools, with their overgrown classes. In the colleges of less notoriety, where the classes are much smaller, the objection does not hold to the same extent.

After disposing of this objection the author asserts that the tendency is towards reformation, and then he states, that by the proper use of the "great engine of modern business—the advertisement," it is possible for the *better*, (often synonymous with *richer*,) schools to crush out the poorer, or to reform them.

Then after a trifle of the pathetic, and a little more congratulation of the profession upon their noble hearts, great souls, distinguished zeal, &c., &c.—and assuring us that our faults are to be all attributed to our great grand-fathers, ("making a stump speech in the belly of the bill,")—he proceeds to enlighten us as to the reforms which are to become inevitable. These are as follows:

Extension of the length of course.

Distribution of the various branches into regular terms appropriated to them.

Diminution of the number of daily lectures.

To all this there can be no sound objection brought, I am confident, that is, if extremes are avoided. In the sketch of the writer's ideal of a medical college, which follows, there is more that is objectionable than he advances. He would have the college undertake the whole instruction of the student upon itself, doing away entirely with all private teaching. His plan is, to have the present course of lectures extended through nine months of the year, only two lectures to be given per day, and twice a week a clinical lecture; each lecture to be preceded by a recitation. The college year is to be divided into two terms, with two vacations of one and two months respectively.

This would preclude all private teaching, except what the student might receive, if he were fortunate enough to obtain a place in a physician's office, while in attendance upon lectures. The result of this cannot be other than the exclusion of the student from the opportunity of observation and study of disease at the bed-side; in short it would eventuate in making didactic teaching the only instruction which the majority of students would receive. The object of this change is, as correctly expressed by the author of the article, to give a larger monopoly of the emoluments and fame of teaching, to the large metropolitan towns of the east; but I think that he labors under a slight mistake in concluding that his plan would secure that result.

As an objection, which he acknowledges may be advanced with some show of reason, he states the unavoidable increase of expense to those who are desirous of entering the medical profession. But this, he says, is not a valid objection. I will let him speak for himself:

"The difficulties in the way will be found in the competition of money-seeking schools, which will cling to the old system and bid for students, on the plea of less time and expense. But let the leading schools of New York and Philadelphia combine in such a plan, and they will soon crush out all opposition. It is hardly to be expected that the movement would be unanimous, but there is much reason to suppose that it would be finally successful. Another and more considerable opposition would arise from the students themselves, or that large class of them who are in search, not of education, but a diploma. But the prestige attending graduation in such a school would do away with this. The argument is money; and those that have money, (I speak here on the supposition that the expenses would be increased, simply by prolonged city residence,) will part with it for the eclat of a thorough education, or for the better motive of the education itself. Those that have no money get through now, with a system nearly as expensive, somehow; and I presume they would do so still. Let them run in debt, or carry along some temporary occupation. Such must oftener beget success than failure; and it is a peculiarity of poor but high-spirited students that they seek the best advantages.

But supposing that it very largely increased the expense of an education, what would be the general result? Men of energy and character find their way into the profession of law through greater difficulties than those with which we propose to environ the entrance to that of medicine. And others than men of energy and character had better stay out. I am aware that the tendency of the day is to cheapen all forms of education, to throw open every pathway to position and preferment. So far as this applies to that degree of education necessary to teach the poor man his rights and his duties to the body politic, all true men will go with this tendency; but this does not reach beyond a comprehensive and liberal system of common schools.

The further disposition to cheapen and make easy the pathway to the learned professions is a specious theory, which flatters only to destroy. It holds out inducements to the weak and indolent to assume the enormous responsibilities of a path of life beyond their abilities; it does them the greatest wrong that can by any possibility be done any man, by placing them in a false position, when a lifetime must be spent in vain regrets over the youthful vanity and folly which led them from the calm and peace of humbler duties to a struggle for which they are incompetent."

Now what a position is this? I cannot doubt that money is a very good thing, but this is the first time that I have known of its being considered synonymous with intellect and ability to become a worthy member of our profession.

The writer says the "disposition to cheapen and make easy the pathway to the learned professions is a specious theory which flatters only to destroy." I have no patience to argue with one holding such opinions; for in him I can only see a man who, favored by fortune with the possession of wealth, is incapable of appreciating other excellence than that which money can purchase. Indeed then!—and poverty is *per se* the evidence of incapacity! Or, if perchance there are just enough exceptions to prove the rule, we are to extend no encouragement to help the less fortunate along towards the knowledge they seek, but advise them to "run in debt," or do some other thing which shall enable them to put more money into the pockets of their aristocratic teachers. I do not know who this "well known lecturer and writer" is who gives utterance to such sentiments, but I would wager a trifle that the young men under his instruction, who are forced to wear seedy garments or forego education, find little encouragement from him. "It is a peculiarity of poor but high spirited students that they seek the best advantages." Well, what follows? Why, take all advantage of this—increase the tariff which they must pay to attain their wishes—build higher the wall which shuts them out from participation in the privileges of the rich—be sure you do not permit the indigent plebeian to trespass upon the high prerogatives of their moneyed betters.

But seriously, sirs: is it not true that the American people owe whatever they possess of superiority over the people of other lands, to the increased facilities for the education of all classes? Is it true that there is anything in *medical knowledge* which makes it the perquisite of those who are, by some lucky accident, rich, and which renders it inappropriate as an acquisition of the poor.

There is a growing tendency, thank God, towards making the privileges of education in all departments of knowledge, free—free to *all*—without money and without price. The North-west leads off in this matter, and I am confident that there is in the movement a soul of good, which will secure it from failure, and although originating from a source which our old fogy brethren of the east affect to despise, I believe they will yet be forced to join in with it or be crushed by it.

I am glad to see by the last number of your journal, that the medical department of our State University has been put upon the list of free schools.

All other reforms which the sense of the profession shall pronounce genuine, I hope to see heartily espoused and fully carried out in the institution.

In concluding this article, already too long, let me say that the evils of which our profession complains, are by no means attributable entirely to our system of education. They are oftener the fruit of *neglect to carry out the system.* And again, the expense now attendant upon acquiring a medical education in our "best schools," is one reason why so many enter upon the practice of medicine, not only without diplomas, but without sufficient study. In France free lectures have not had the effect to lower the standard of professional knowledge or of demoralizing the profession. I am not yet prepared to acknowledge that our own people are so much more degenerate than the French as to make it dangerous to the interests of science to render the acquisition of knowledge easy to all.

My ideal of a system of medical education has, as one of its most important elements, entire abolition of fees from the student—the teacher receiving the reward of his labor from endowment of colleges by state governments or otherwise—and then a legal prohibition to practice without a full compliance with the requirements of the colleges as to education.

Yours &c., W.

